# Ovarian metastasis from nongynecologic primary sites: a retrospective analysis of 177 cases and 13-year experience

**DOI:** 10.1186/s13048-020-00714-8

**Published:** 2020-10-27

**Authors:** Jing-Jing Zhang, Dong-Yan Cao, Jia-Xin Yang, Keng Shen

**Affiliations:** 1 Department of Obstetrics and Gynecology, Peking Union Medical College Hospital, Chinese Academy of Medical Science and Peking Union Medical College, No. 1 ShuaiFuYuan, Dongcheng District, Beijing, 100730 China; 2 Department of Gynecology, Sun Yat-sen First Affiliated Hospital, No. 58 Zhongshan 2nd Road, Yuexiu District, Guangzhou, 510080 China

**Keywords:** Ovarian metastasis, Nongynecologic primary site, Cytoreductive surgery, Residual lesion, Differentiation, Prognosis

## Abstract

**Background:**

Metastasis to the ovary from nongynecologic organs accounts for 9% of all ovarian malignancies. Although the most common nongynecologic primary site of ovarian metastasis is the gastrointestinal tract, metastasis from other sites to the ovary is not uncommon. Differential diagnosis of primary and metastatic ovarian tumors is important; otherwise, appropriate treatment cannot be determined. Furthermore, an optimal treatment strategy for ovarian metastasis from nongynecologic primary sites still needs to be explored.

**Methods:**

One hundred seventy-seven patients with ovarian metastasis from nongynecologic primary sites admitted to Peking Union Medical College Hospital between May 2005 and May 2018 were retrospectively evaluated.

**Results:**

The mean age was 48 years (range, 18–83). Approximately 60% of patients were premenopausal women. The two most common nongynecologic primary sites of ovarian metastasis were the colorectum (68 cases) and stomach (61 cases). In addition to the most common symptoms of abdominal distension (39.0%), abdominal pain (37.9%), and ascites (27.7%), 18.1% of patients presented with abnormal uterine bleeding. Half of the patients who tested serum CA-125 preoperatively had elevated CA-125 levels within the range of 35 U/ml to 200 U/ml. More than 70% of synchronous ovarian metastases were preoperatively misdiagnosed as primary ovarian cancer. Of all included cases, 56.5% achieved optimal cytoreductive surgery (the diameter of the largest residual lesion < 2 cm). The overall 5-year survival rate and median survival time were 10% and 20 months, respectively. The primary site, optimal cytoreductive surgery, tumor differentiation, and postoperative adjuvant treatment were identified as prognostic indicators.

**Conclusions:**

The colorectum and stomach are the most common nongynecologic primary sites of ovarian metastasis. Synchronous ovarian metastasis is easily misdiagnosed as primary ovarian cancer. Optimal cytoreductive surgery and postoperative adjuvant treatment can be performed to confer survival benefit in selected patients.

## Background

As in the lung and liver, the ovary is a frequent site of metastasis for genital and nongynecologic primary malignancies [1–3]. The gastrointestinal tract is the most common nongynecologic primary tumor site that metastasizes to the ovaries, followed by the breast [1, 2].

Inappropriate treatment and adverse outcomes may occur when ovarian metastasis and primary ovarian cancer are misdiagnosed as each other [4]. Optimum surgical debulking is the most important treatment strategy for ovarian primary cancer because minimal residual lesion after surgery is associated with prolonged survival [5]. However, an optimal treatment strategy for ovarian metastasis from nongynecologic primary sites has not been established. First, Akhan S. E. et al. [6] suggested that aggressive surgery should be avoided in patients with extragenital metastatic cancers of the ovary, especially in those complicated by peritoneal metastasis. The presence of distant metastatic sites, including the ovary, usually indicates a dismal prognosis. Goere D. et al. [7] found that ovarian metastasis was less responsive to chemotherapy, so they proposed that ovarian metastasectomy should always be considered, even in cases of nongynecologic metastases.

The purpose of our study was to evaluate the demographic characteristics, disease presentation, role of surgery and postoperative adjuvant treatment, survival and its prognostic indicators.

## Methods

### Study population

One hundred seventy-seven patients with ovarian metastasis from nongynecologic primary sites underwent surgery at Peking Union Medical College Hospital (PUMCH) between May 2005 and May 2018. Ethics approval was granted by the Institutional Review Board of PUMCH. Clinicopathologic information was obtained from medical records and pathology reports. Follow-up information was obtained from telephone interviews. Survival was defined as the date of resection of ovarian metastasis to the date of death or to May 31, 2018. The following data were collected: (1) the general conditions; (2) presenting symptoms of ovarian masses related, serum carbohydrate antigen-125 (CA-125), preoperative imaging examination; (3) the primary sites, the date of diagnosis of primary cancer, the date of resection of ovarian metastasis, and the interval between the two dates; (4) preoperative diagnosis of ovarian mass; (5) surgical findings, residual lesion size at the completion of surgery, surgical complications; (6) postoperative pathology reports; (7) any postoperative adjuvant treatment administered; and (8) survival time. Patients with incomplete clinical-pathological data were excluded from our study.

The chronological sequence of diagnosis was identified as synchronous or metachronous according to the diagnostic date of primary cancer and ovarian metastasis. Namely, the patients with a previous history of nongynecologic primary cancer before detection of the ovarian metastasis were assigned to the metachronous group; otherwise, they were assigned to the synchronous group. The interval between the two dates in the metachronous group is calculated.

All enrolled patients underwent surgery in our hospital. According to the surgical findings, extraovarian involvement was determined as positive or negative. In consideration of the lack of a uniform definition of radical or palliative surgery and the diverse extent of surgical removal, we focused on the residual lesion size at the completion of surgery. Surgery was considered optimal if the diameter of the largest residual lesion was less than 2 cm.

All ovarian masses had pathological confirmation of the surgical specimens as ovarian metastasis. According to the pathological results, the degree of differentiation was determined to be well differentiated, moderately differentiated, poorly differentiated and undifferentiated. A majority of primary sites were determined by previous surgical pathology at our hospital or other hospitals. The rest of the enrolled patients had neither pathology nor diagnostic imaging examination to identify the primary sites. These were classified as the group of unknown primary sites.

### Statistical analysis

The data were analyzed using SPSS for IOS, version 21. Survival rates were calculated using the life table method, and differences between groups were calculated using the log-rank test. A two-sided *P* value of 0.05 or less was regarded as statistically significant.

## Results

### Patient characteristics, presenting symptoms, preoperative examinations

Between May 2005 and May 2018, 177 female patients underwent surgery for ovarian metastasis from nongynecologic primary sites at our center. The mean age was 47.6 ± 13.2 years (range, 18–83), and 59.3% were premenopausal. The most common presenting symptoms were abdominal pain (67/177, 37.9%), abdominal distension (69/177, 39%), ascites (49/177, 27.7%), and abnormal uterine bleeding (32/177, 18.1%) (Table 1).

**Table 1:** Patient characteristics of the 177 patients

Characteristics	N(%)
Age (mean ± SD, years)	47.6 ± 13.2
Menopausal status	
Premenopausal	105 (59.3%)
Postmenopausal	72 (40.7%)
Presenting symptoms	
Abdominal pain	67 (37.9%)
Abdominal distension	69 (39.0%)
Ascites	49 (27.7%)
Abnormal uterine bleeding	32 (18.1%)
Preoperative serum CA-125	
Not measured	23 (13.0%)
Normal	38 (21.5%)
Abnormal (< 200 U/ml)	77 (43.5%)
Abnormal (≥ 200 U/ml)	39 (22.0%)
Preoperative imaging examination	
Positive	174 (98.3%)
Negative	3 (1.7%)

The preoperative CA-125 value was tested in 116 patients. Serum CA-125 was elevated (> 35 U/ml) in 65.5% of these patients. Of the patients with elevated CA-125, the majority (77/116, 66.4%) had a slight increase in CA-125 (< 200 U/ml) (Table 1).

Ovarian mass was found in 98.3% of the patients by preoperative imaging examination (Table 1).

### Preoperative diagnosis and postoperative pathology

Among our patients, the primary cancers were located in the colorectum (68/177, 38.4%), stomach (61/177, 34.5%), appendix (12/177, 6.8%), biliary tract (7/177, 4.0%), pancreas (7/177, 4.0%), breast (6/177, 3.4%), small intestine (5/177, 2.8%), lung (3/177, 1.7%), bladder (2/177, 1.1%), and unknown sites (6/177, 3.4%) (Table 2).

**Table 2:** Interval of metachronous ovarian metastasis and differentiation of tumors of the 177 patients

	Colorectum (*n* = 68)	Stomach (*n* = 61)	Appendix (*n* = 12)	Biliary Tract (*n* = 7)	Pancreas (n = 7)	Breast (n = 6)	Small intestine (*n* = 5)	Lung (*n* = 3)	Bladder (*n* = 2)	Unknown^a^ (n = 6)
Chronological sequence Synchronous	40 (58.8%)	30 (49.2%)	10 (83.3%)	2 (28.6%)	4 (57.1%)	–	2 (40.0%)	1 (33.3%)	1 (50.0%)	6 (100.0%)
Metachronous	28 (41.2%)	31 (50.8%)	2 (16.7%)	5 (71.4%)	3 (42.9%)	6 (100.0%)	3 (60.0%)	2 (66.7%)	1 (50.0%)	–
Median interval^b^ (range, months)	12.5 (2–40)	20 (1.5–240)	95, 95	51 (12–73)	112 (19–115)	54 (7–81)	18 (12–34)	4, 48	135	–
Differentiation Well	10 (14.7%)	–	4 (33.3%)	–	3 (42.9%)	–	–	–	–	–
Moderate	42 (61.8%)	6 (9.8%)	4 (33.3%)	3 (42.9%)	2 (28.6%)	–	2 (40.0%)	–	1 (50.0%)	2 (33.3%)
Poor/undifferentiated	16 (23.5%)	52 (85.2%)	4 (33.3%)	1 (14.3%)	1 (14.3%)	5 (83.3%)	3 (60.0%)	3 (100.0%)	0 (0%)	2 (33.3%)
Not available	0 (0%)	3 (4.9%)	–	3 (42.9%)	1 (14.3%)	1 (16.7%)	–	–	1 (50.0%)	2 (33.3%)

^a^ The patients had neither pathology nor diagnostic imaging examinations to identify the primary cancers who were classified as the group of unknown primary sites

^b^ If the number of cases is less than 3, then the intervals are listed.

Ninety-six patients (54.2%) presented with synchronous ovarian metastasis, while 81 (45.8%) developed metachronous ovarian metastasis after the diagnosis of primary cancers. In metachronous cases, the median interval was 19 months (range, 1.5–240 months) (Table 2).

Tumor differentiation could be identified in 166 patients. Of them, well-differentiated tumors accounted for 9.6%, moderately differentiated tumors for 35%, poorly differentiated tumors for 49.2% and undifferentiated tumors for 6.2% (Table 2).

According to the clinical manifestations and previous history, a total of 103 patients (58.2%) were tentatively diagnosed with ovarian metastatic tumors before surgery. Twenty-six patients were from the synchronous group, and 77 patients were from the metachronous group. A significant difference between the two groups was observed in terms of preoperative diagnostic accuracy (*P* <  0.0001) (Table 3).

**Table 3:** Preoperative diagnostic accuracy in the metachronous and synchronous groups

	Tentatively diagnosed with ovarian metastasis		Diagnostic accuracy	*P*-value
	Yes	No		
Synchronous group	26	70	27.1%	< 0.0001
Colorectum	14	26		
Stomach	8	22		
Appendix	0	10		
Small intestine	0	2		
Biliary tract	0	2		
Lung	0	1		
Pancreas	3	1		
Bladder	0	1		
Unknown	1	5		
Metachronous group	77	4	95.1%	

### Surgery and postoperative adjuvant treatment

According to the surgical findings, 54.8% of patients had bilateral ovarian involvement, and 61.6% of patients had extraovarian involvement. At the completion of surgery, the largest residual lesion less than 2 cm was achieved in 100 cases (56.5%) (Table 4).

**Table 4:** Surgery and postoperative adjuvant therapy of the 177 patients

	N(%)
Bilaterality	
Bilateral	97 (54.8%)
Unilateral	80 (45.2%)
Median tumor diameter (range, cm)	8.5 (1.0–35.0)
Extraovarian involvement	
Positive	109 (61.6%)
Negative	68 (38.4%)
Residual lesion	
< 2 cm	100 (56.5%)
≥ 2 cm	77 (43.5%)
Surgical complications	
Yes	17 (9.6%)
No	160 (90.4%)
Adjuvant treatment	
Yes	112 (63.3%)
No	65 (36.7%)

Surgical complications occurred in approximately 10% of patients (17/177) and included anemia, infection, intestinal obstruction, poor wound healing, and cardiovascular-related adverse events. One patient died of pulmonary embolism (Table 4).

After surgery, 112 (63.3%) patients received adjuvant chemotherapy and/or radiation based on the primary cancers based on the primary site (Table 4).

### Survival

**Fig. 1:**
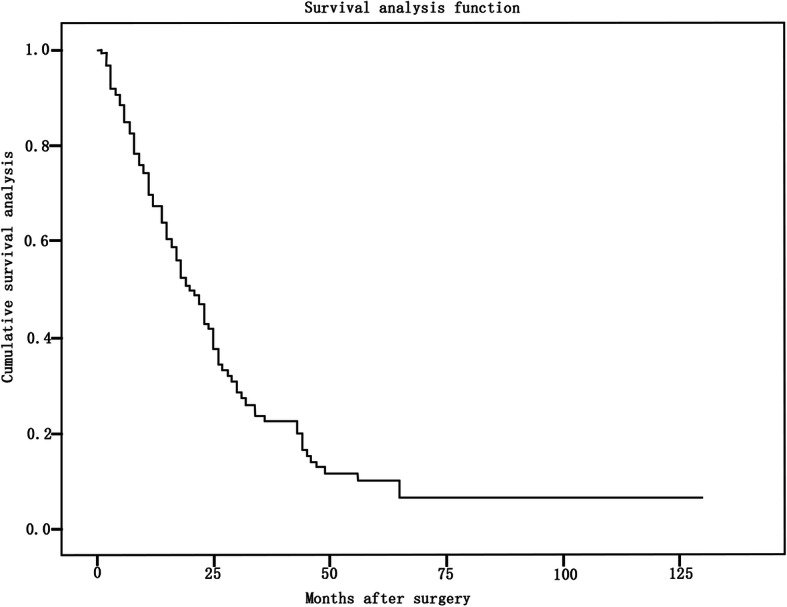
Overall survival of 177 patients. The overall 3-year survival rate and 5-year survival rate were 23 and 10%, respectively, and the median survival time was 20 months (95% CI, 16–24 months)

The overall 3-year survival rate and 5-year survival rate were 23 and 10%, respectively, and the median survival time was 20 months (95% CI, 16–24 months). (Fig. 1).

**Fig. 2:**
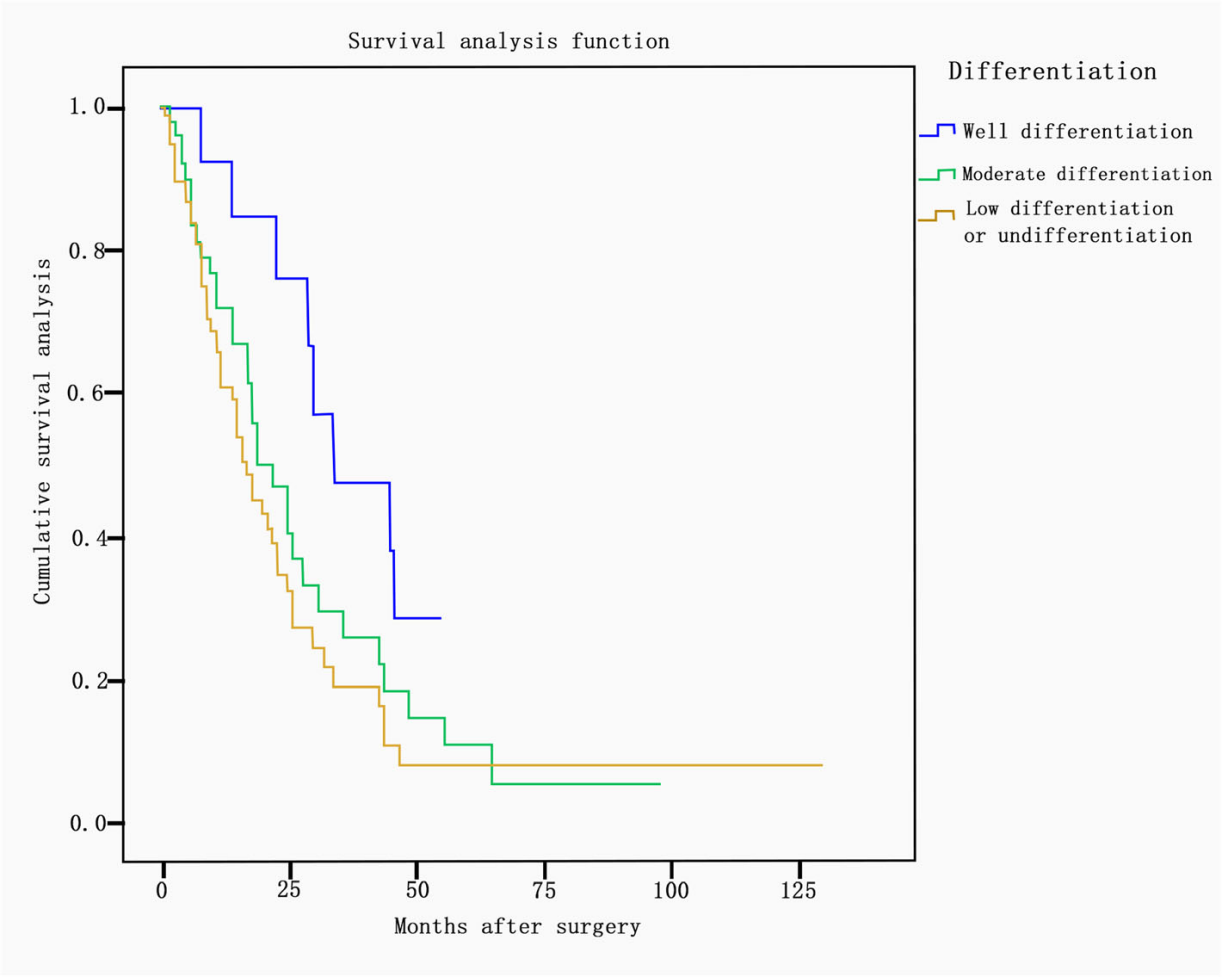
Survival according to differentiation (well-differentiated, 34 months; moderately differentiated, 21 months; poorly differentiated or undifferentiated, 16 months). The median survival times according to the differentiation of tumors showed significant differences (*P* = 0.016)

**Fig. 3:**
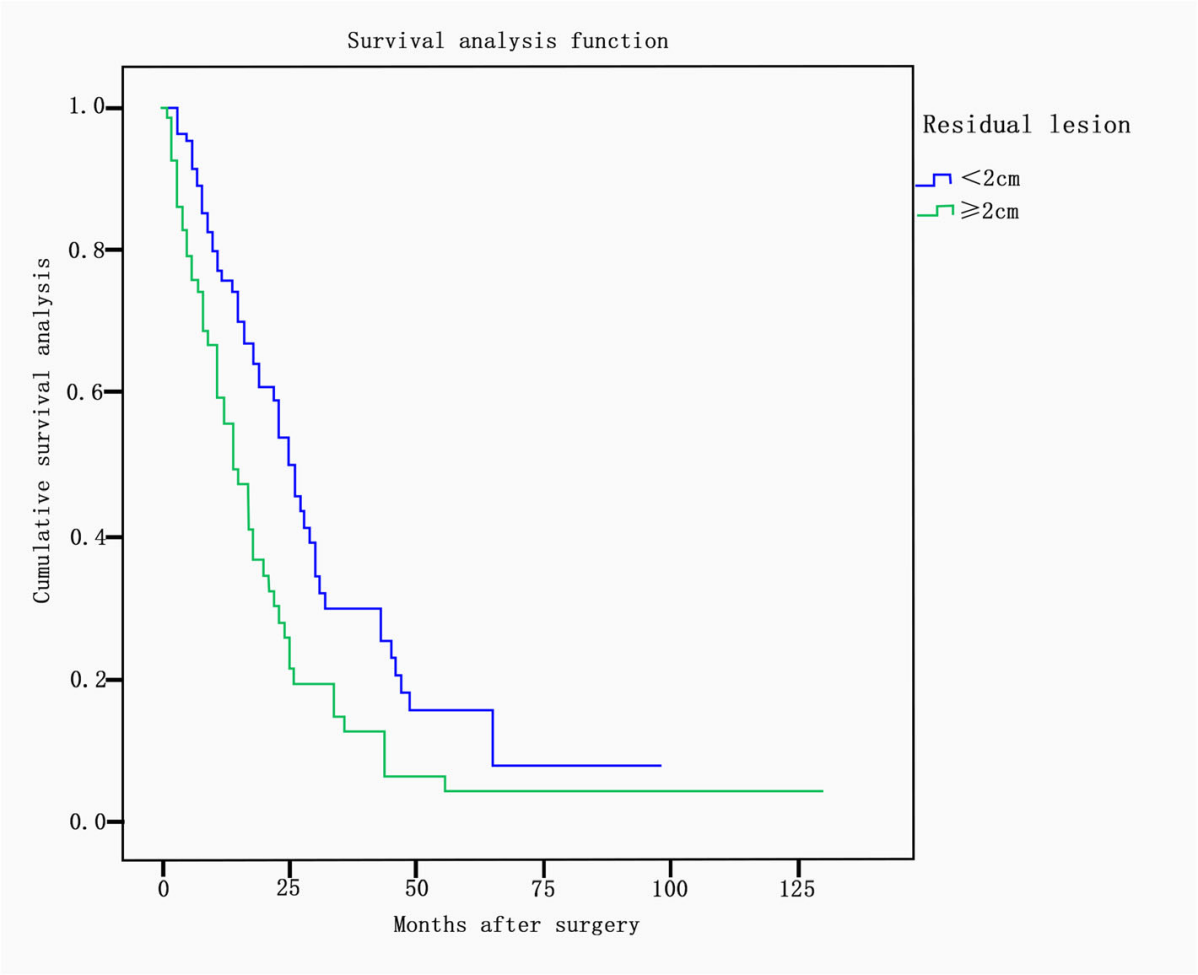
Survival according to the residual lesion (< 2 cm or ≥ 2 cm). The median survival time was 25 months for patients whose largest residual lesion was less than 2 cm and 14 months for those whose largest residual lesion was more than or equal to 2 cm (*P* = 0.001)

**Fig. 4:**
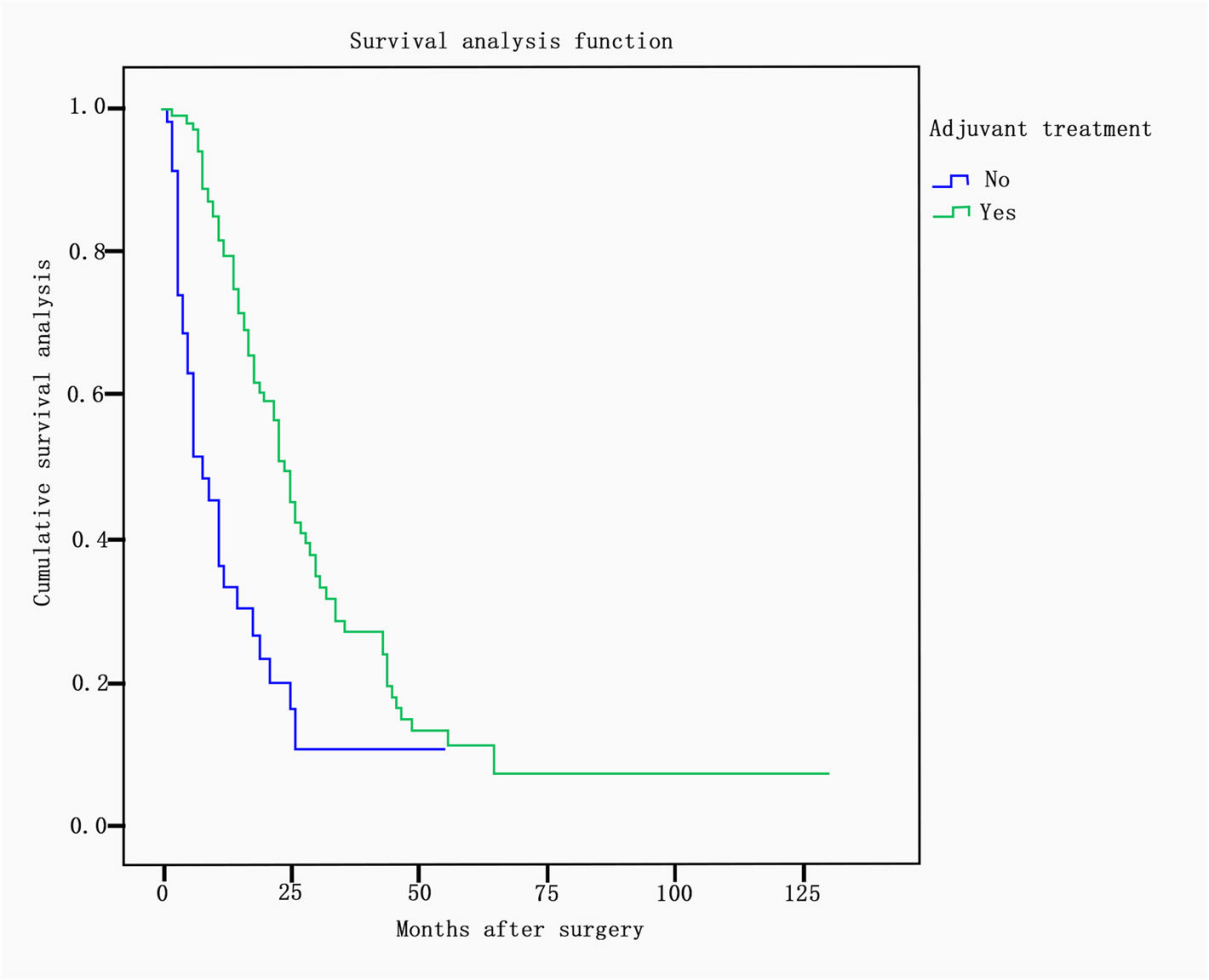
Survival according to postoperative adjuvant treatment. There was a significant difference in survival between patients who received postoperative adjuvant treatment and those who did not, with an estimated median survival of 24 months and 8 months, respectively (*P* < 0.001). The four figures included in this manuscript were drawn by JJ-Z, the first author of the manuscript. The other authors of this manuscript agree with the four figures. The four figures have not been published previously. All the authors agree to give the *Journal of Ovarian Research* permission to publish the four figures

There was a significant difference in the median survival time between different primary sites (*P* = 0.02). The median survival times according to primary site were as follows: small intestine, 59 months; appendix, 54 months; bladder, 27 months; breast, 25 months; lung, 25 months; colorectum, 21 months; stomach, 18 months; biliary tract, 14 months; and pancreas, 13 months. The median survival times showed significant differences according to tumor differentiation (*P* = 0.016) and were as follows: well-differentiated, 34 months; moderately differentiated, 21 months; and poorly differentiated/undifferentiated, 16 months (Fig. 2). The median survival time was 25 months in patients whose largest residual lesion was less than 2 cm and 14 months in those whose largest residual lesion was more than or equal to 2 cm (*P* = 0.001) (Fig. 3). In terms of adjuvant treatment, there was a significant difference in survival between patients who received postoperative chemotherapy and/or radiation and those who did not, with estimated median survival times of 24 months and 8 months, respectively (*P* <  0.001) (Fig. 4). However, age, menopausal status, presence of ascites, CA-125 level (< 200 U/ml vs. ≥ 200 U/ml), bilaterality of ovarian metastasis, extraovarian involvement, and chronological sequence of diagnosis (synchronous vs. metachronous) did not affect survival.

## Discussion

The colorectum (68/177, 38.4%) and stomach (61/177, 34.5%), both belonging to the gastrointestinal tract, were the first two common nongynecologic primary sites of ovarian metastasis in the current study. Generally, it is consistent with the study of Kim W. Y. et al. [8] from Korea. Their study showed that the primary sites were mainly the stomach (73/158, 46.2%) and colon (61/158, 38.6%). Another study by Yada-Hashimoto N. et al. [1] from Japan reported that the stomach (15/38, 39.5%), breast (9/38, 23.7%), and colon (7/38, 18.4%) were the top three primary sites of nongynecologic organs. Obviously, because gastric cancer is more common in East Asian populations, the stomach is the nongynecologic primary site of ovarian metastasis. Outside this region, the most common nongynecologic primary site of ovarian metastasis may be different. A related study from Turkey showed that the breast (35/154, 22.7%) and stomach (35/154, 22.7%) were tied for first place [9]. Two American series reported that the breast was the primary site [2, 10]. In our study, the primary sites were the colorectum, stomach, appendix, biliary tract, pancreas, breast, small intestine, lung, and bladder. According to other published studies, renal pelvis [10], lymph system [1, 9], melanoma of the skin [2], mesothelioma [9, 10], and thyroid carcinoma [11] can also be the nongynecologic primary sites of ovarian metastasis. Awareness of the primary sites of ovarian metastasis helps clinicians differentiate primary from metastatic ovarian cancer as well as search for a possible primary site purposefully.

The mean age of our patients was 48 years when they were diagnosed, approximately 10 to 15 years younger than the age of primary ovarian cancer (late 50s to early 60s) [5]. A high proportion of our patients were premenopausal women (59.3%), and the increased blood flow to the ovaries observed in premenopausal women is considered a contributing factor in ovarian metastasis [12].

In our study, the top three symptoms were abdominal distension (39.0%), abdominal pain (37.9%), and ascites (27.7%). These atypical symptoms are similar to the presenting symptoms reported in the studies by Ayhan A. et al. and Kim W. Y. et al., including distension/pressure symptoms, palpable/abdominopelvic masses, and abdominal pain; in addition, a small group of patients in their studies was asymptomatic [8, 9]. One reason for the difficulty in differentiating primary from metastatic ovarian cancer is that their presenting symptoms can overlap. For primary ovarian tumors, presentation with 3 to 4 months of abdominal distension or pain is typical [5]. Furthermore, abnormal uterine bleeding occurred in 18.1% of our patients. Li-chun L. et al. [13] reported that 14.3% of their patients with Krukenberg tumors experienced menstrual irregularity. One study mentioned above showed that only 5.2% of their patients presented abnormal uterine bleeding [9]. In addition, there is a case report on postmenopausal vaginal bleeding as the initial presentation of Krukenberg tumor [14]. Abnormal uterine bleeding is considered to be caused by the disruption of ovarian function and effect on sex hormone levels due to ovarian metastasis.

Among our patients who tested serum CA-125 before surgery, approximately half of the patients had an abnormally elevated CA-125 of more than 35 U/ml but no more than 200 U/ml. Serum CA-125 is the most common tumor marker for ovarian cancer with low specificity and sensitivity [15]. Clinically, increases in CA125 levels of up to several thousand times is relatively common in epithelial ovarian cancer, while it is not frequently observed in metastatic ovarian cancer. The absolute level of CA-125 may help to differentiate primary from metastatic ovarian cancer. Although many gastrointestinal cancer tumor markers, such as CA-19-9, CEA, and CA-72-4, also have low sensitivity and specificity [16, 17], these elevated tumor markers could serve as a preoperative clue for clinicians. Additionally, tumor markers with differential diagnostic value include CA199, which is often elevated in pancreatic cancer, and CA153, which is often elevated in breast cancer. Unfortunately, these tumor markers are not as commonly tested as CA-125 in patients with ovarian masses.

Patients in the metachronous group accounted for 45.8% of the total patients in our study. These patients had a primary cancer in the past and then developed ovarian metastasis with a median interval of 19 months (range, 1.5–240 months). Different primary tumors showed different variations in terms of their metastasis interval: ovarian metastasis from bladder cancer, pancreatic cancer and appendix cancer often occurs approximately 10 years after the primary tumors; that from biliary tract cancer, breast cancer and lung cancer occurs in 4 to 5 years; and that from stomach cancer, small intestine cancer, and colorectal cancer occurs in 1 to 2 years. Kim W. Y. et al. [8] showed that their stomach cancer patients and colon cancer patients developed ovarian metastasis at median times of 15.5 months and 13.5 months, respectively; their three breast cancer patients developed ovarian metastasis within 41, 47, and 73 months. Taranto A. J. et al. [18] reported a case of ovarian metastasis arising from gall bladder carcinoma with a diagnosis of 4 years previously. Two cases of nonfunctional neuroendocrine carcinoma of the pancreas developed ovarian metastasis 5 and 7 years after diagnosis, respectively [19, 20]. These findings are nearly consistent with ours. However, studies on non-small-cell lung cancer patients have reported an interval of 14 to 20 months [21, 22], which seems to be a shorter duration than ours. Considering that this may be related to the pathological type of lung cancer, we further confirmed that the pathological type of all our enrolled lung cancer patients was small-cell lung cancer. Generally, non-small-cell lung cancer has a higher degree of malignancy and poorer prognosis than small-cell lung cancer, which explains why our metachronous lung cancer patients had a longer interval. In addition, out of the primary sites of our study, Corrado G. et al. [11] reported a rare case of ovarian metastasis from thyroid carcinoma 9 years after diagnosis. Over time, after a diagnosis of nongynecologic primary cancer, clinicians must be mindful of the development of ovarian metastasis. It is valuable not only because ovarian metastatic tumors are relatively common but also because familiarity with the probable intervals for different primary cancers is helpful for clinicians to tentatively diagnose ovarian metastasis.

Indeed, 58.2% of our patients were tentatively diagnosed with ovarian metastasis before surgery. The preoperative diagnostic accuracy was 95.1 and 27.1% in the metachronous and synchronous groups, respectively. A significantly lower accuracy in the synchronous group indicates that synchronous ovarian metastasis misdiagnosed as primary ovarian cancer is quite common. Previous history is a strong indication for the correct diagnosis of ovarian metastases, which greatly contributes to the high preoperative diagnostic accuracy of metachronous group. In the synchronous group, preoperative diagnosis could only be comprehensively considered and determined based on clinical manifestations, physical examination, preoperative imaging examination, and some serum tumor markers with low specificity and sensitivity, which leads to the low preoperative diagnostic accuracy. Patients with synchronous ovarian metastasis from the appendix, small intestine, biliary tract, lung, and bladder were all diagnosed incorrectly before surgery. Of synchronous ovarian metastases, 65% from the colorectum and 73.3% from the stomach were misdiagnosed as primary ovarian cancer. Furthermore, and of particular note, no breast cancer patients had synchronous ovarian metastasis in our study, which may be related to the early detection of breast cancer. Unfortunately, little research has been conducted to analyze synchronous ovarian metastasis mimicked by primary ovarian cancer. Without considering the chronological sequence of diagnosis, based on the data available in the literature, 32% of mimicking metastases were from the colorectum, and 49% of these were from the gastrointestinal tract [23, 24]. Although the differentiation of synchronous ovarian metastasis and primary ovarian cancer is a difficult problem, it has yet to be resolved. Keeping the possibility of ovarian metastasis in mind, patients presenting ovarian masses of unknown origin should undergo an extensive search to rule out suspicious primary sites by fully utilizing various imaging examinations, gastrointestinal endoscopy, serum CA-125 and other biomarkers. However, if these methods cannot help to confirm the diagnosis, surgical resection and pathology of the ovary should be a last resort.

The purpose of surgical intervention is not only to establish a definite diagnosis but also to alleviate symptoms, minimize residual tumor burden, avoid progression or achieve a cure. Usually, surgeons proceed with surgical interventions depending on the presence of severe symptoms or emergencies or if the surgical removal of tumors is deemed necessary for asymptomatic patients following a multidisciplinary team consensus. Due to the variety of the tumor size, depth of invasion, and involvement, the extent of surgical removal varied; the minimal extent was only unilateral salpingo-oophorectomy, and the maximal extent was resection of multiple involved organs and local lymph nodes. Over the past decade, a number of retrospective studies have proven that the residual lesion size at the completion of surgery is related to a survival benefit for patients with ovarian metastasis from nongynecologic primary cancers [8, 9, 25–32]. A cut-off value of less than 2 cm residual lesion for assessing the effect of cytoreductive surgery has been long-used among the literatures cited above [8, 25, 32]. In the current study, there was a significant survival difference between patients with less than 2 cm of the largest residual lesion and those with more than or equal to 2 cm of the largest residual lesion. The median survival times of the two groups were 25 months and 14 months, respectively. This result suggests that every effort to perform optimal surgery should be made. However, the underlying surgical complications must be evaluated before surgery. Surgeons made the decision to proceed with surgery after balancing surgical benefits and risks. In our series, although approximately 10% of patients experienced complications, fatal complications were observed in only one patient, which is consistent with the results of Seow-En I [33].. Generally, surgery for patients with ovarian metastasis from nongynecologic primary cancers can be performed safely with an acceptable complication rate.

The prognosis of patients with ovarian metastasis from nongynecologic primary sites is poor. The published study of the largest sample size (158 patients) reported that the 5-year survival rate and median survival time were 7.2% and 15 months, respectively [8]. These results were not different from ours. In our study, the 3-year survival rate, 5-year survival rate, and median survival time were 23, 10%, and 20 months, respectively. In addition to the residual lesion size, our results showed that primary sites, the differentiation of ovarian metastasis and postoperative adjuvant treatment were also prognostic indicators.

A considerable number of studies have reported that the survival of patients with ovarian metastasis according to the primary tumor showed significant differences [1, 2, 6, 8, 9, 25, 29, 34]. Although the primary sites included in each study were different, among the included common nongynecologic primary cancers, the overall survival time of breast cancer was longer than that of gastrointestinal cancer, and the overall survival time of colorectal cancer was longer than that of gastric cancer. In our study, the median survival times of breast cancer, colorectal cancer and stomach cancer were 25 months, 21 months and 18 months, respectively, consistent with the results of the aforementioned earlier studies. Moreover, for the reason of a greater variety of nongynecologic primary sites included in our study than other studies, some cancers with relatively good prognosis, such as appendix mucinous adenocarcinoma and bladder transitional cell carcinoma, survive longer after resection of ovarian metastasis, whose median survival times were 54 months and 27 months, respectively. In contrast, in our study, patients with biliary tract and pancreatic cancers and a poorer prognosis died one year after surgery for ovarian metastasis. All 5 enrolled cases of small intestinal cancer were adenocarcinoma. According to the literature, the prognosis of small intestinal adenocarcinoma at an intermediate stage seems similar to that of colon and gastric cancers [35]. The 59-month median survival time of small intestine cancer after surgery was the longest in our study, which suggests that prognosis is not entirely consistent with postoperative survival time for ovarian metastasis. Due to the lack of relevant studies, this phenomenon cannot be explained at present.

To the best of our knowledge, no study has specifically analyzed the differentiation of ovarian metastasis as an underlying prognostic indicator. Nevertheless, our results showed that the median survival times of patients with well-differentiated, moderately differentiated, poorly differentiated or undifferentiated ovarian metastases were significantly different—34 months, 21 months, and 16 months, respectively.

Postoperative adjuvant treatment is regarded as essential. The results of both an early study [8] and our current study have proven that patients who undergo postoperative adjuvant treatment, mainly chemotherapy, survive longer than those who do not.

According to the aforementioned prognostic indicators, patients with ovarian metastasis from nongynecologic primary sites can be selected to undergo optimal cytoreductive surgery, which has a low rate of surgical complications and confers survival benefits to patients.

## Conclusion

Surgery should be considered for patients with ovarian metastasis from nongynecologic primary sites. The decision to proceed with cytoreductive surgery can be affected by the presence of symptoms, the primary sites, and the level of tumor differentiation. The surgery needs to be completed in an experienced hospital by a multidisciplinary team composed of gynecology department, the corresponding department responsible for the primary cancer, oncology department and surgery department. Every effort should be made to achieve optimal cytoreductive surgery, namely, the diameter of the largest residual lesion at the completion of surgery is less than 2 cm, followed by postoperative adjuvant treatment.

## Data Availability

The dataset used or analyzed in this study is available from the co-corresponding authors upon reasonable request.

## Abbreviations

PUMCH: Peking Union Medical College Hospital
CA-125: Carbohydrate antigen-125
CA19–9: cancer antigen 19–9
CA72–4: cancer antigen 72–4
CEA: Carcinoembryonic antigen
